# Synthesis and Characterization of Nonwoven Cotton-Reinforced Cellulose Hydrogel for Wound Dressings

**DOI:** 10.3390/polym13234098

**Published:** 2021-11-25

**Authors:** Faheem Ahmad, Bushra Mushtaq, Faaz Ahmed Butt, Muhammad Sohail Zafar, Sheraz Ahmad, Ali Afzal, Yasir Nawab, Abher Rasheed, Zeynep Ulker

**Affiliations:** 1 School of Engineering & Technology, National Textile University, Faisalabad 37610, Pakistan; faheem@ntu.edu.pk (F.A.); bushrantu@gmail.com (B.M.); aliafzal@ntu.edu.pk (A.A.); ynawab@ntu.edu.pk (Y.N.); abher.rasheed@ntu.edu.pk (A.R.); 2 Materials Engineering Department, NED University of Engineering and Technology, Karachi 75270, Pakistan; faazbutt@neduet.edu.pk or; 3 Department of Restorative Dentistry, College of Dentistry, Taibah University, Al Madinah Al Munawwarah 41311, Saudi Arabia; 4 Department of Dental Materials, Islamic International Dental College, Riphah International University, Islamabad 44000, Pakistan; 5 School of Pharmacy, Altinbas University, Istanbul 34147, Turkey; zeynep.ulkerdemir@altinbas.edu.tr

**Keywords:** hydrogels, nonwoven, cellulose, wound dressing

## Abstract

Hydrogels wound dressings have enormous advantages due to their ability to absorb high wound exudate, capacity to load drugs, and provide quick pain relief. The use of hydrogels as wound dressings in their original form is a considerable challenge, as these are difficult to apply on wounds without support. Therefore, the incorporation of polymeric hydrogels with a certain substrate is an emerging field of interest. The present study fabricated cellulose hydrogel using the sol–gel technique and reinforced it with nonwoven cotton for sustainable wound dressing application. The nonwoven cotton was immersed inside the prepared solution of cellulose and heated at 50 °C for 2 h to form cellulose hydrogel–nonwoven cotton composites and characterized for a range of properties. In addition, the prepared hydrogel composite was also loaded with titania particles to attain antibacterial properties. The Fourier transform infrared spectroscopy and scanning electron microscopy confirmed the formation of cellulose hydrogel layers inside the nonwoven cotton structure. The fabricated composite hydrogels showed good moisture management and air permeability, which are essential for comfortable wound healing. The wound exudate testing revealed that the fluid absorptive capacity of cellulose hydrogel nonwoven cotton composite was improved significantly in comparison to pure nonwoven cotton. The results reveal the successful hydrogel formation, having excellent absorbing, antimicrobial, and sustainable properties.

## 1. Introduction

Traditional wound dressings such as bandages, gauze, and cotton pads [1] are often used to cover the wound [2]. These dry dressings lack antibacterial activity and are not efficient in terms of absorbing wound exudate and relieving pain, therefore likely to delay the wound healing process [3,4]. Moreover, these dressings adhere to the wound through sticky exudate, which debrides the wound and damages the granulation tissues upon removal from skin [5]. In the recent era, wound dressings are not used only for wound covering but also for accelerating the wound healing process. Numerous current research studies revealed that a moist environment is needed to accelerate the wound healing process [6,7]. Based on this requirement, wet wound dressings have been developed, which provide a moist environment, accelerate the wound healing process and skin repair, and avoid scar formation [2,8]. In the light of these requirements, the hydrogel-based wound dressing is an emerging field in the area of wound management.

Hydrogels are 3D crosslinked polymeric gels that have the capacity to trap a large amount of water [9]. The hydrophilic crosslinked polymer and water are the two components of the hydrogel network [10]. Hydrogels have the ability to absorb and hold large amounts of water while maintaining their three-dimensional structure and physical shape [11,12]. Owing to the the large water absorption capacity, high swelling characteristics, good biocompatibility, and biodegradability [13,14] hydrogels have numerous applications in the field of agriculture [15,16,17], food [18,19,20], cosmetics [21,22], biomedical [23,24], tissue regeneration [25,26], and wound dressings [27,28]. The hydrogel-based wound dressings have direct contact with the wound, so they must exhibit biocompatible, biodegradable, and nontoxic properties [2,29,30,31]. Hydrogels are commonly prepared from organic materials such as alginate, starch, chitosan, and cellulose. Microcrystalline cellulose (MCC) synthesized from α-cellulose precursors are used in pharmaceutical applications owing to their functional properties [32]. MCC is a biocompatible and biodegradable polymer, with high water-absorbing properties [33,34,35,36]. Moreover, MCC is the derivative of cellulose that is an abundantly available natural polymer with a tight molecular chain structure and is stabilized with hydrogen bonding [37]. Cellulose-based hydrogels have various applications in the area of wound care due to the increasing demand for environmentally friendly and sustainable products [38,39,40].

The resistance against microbes is another advantage of modern wound dressings. Several metal oxides have antibacterial properties that have drawn increasing attention in the medical field. Among these metal oxides, titanium oxide (TiO_2_) has considerable potential in the biomedical field [41,42]. TiO_2_ has the ability to destroy bacteria, viruses, and even cancer cells [43,44]. Moreover, it is a nontoxic, biocompatible, and biologically inert substance for humans. Therefore, TiO_2_ particles have the potential to be incorporated into the hydrogel structure for wound dressing applications [45]. Biomedical hydrogel-based wound dressings with antibacterial properties exhibit many desirable properties, but they are difficult to grip on the wound area [2,46]. Hydrogels do not have the desired strength and flexibility, which causes their slippage and breakage from the wound. Therefore, there is a need for supporting material that provides stability and shape to a hydrogel-based wound dressing. Textile fabrics (woven and nonwoven) are one of the best substrates to incorporate with hydrogel without affecting the functional characteristics of the hydrogel-based wound dressings [47].

Nonwoven fabrics are soft, lightweight, highly porous, and easy to manufacture with less cost among the available textile substrates. Nonwoven fabrics are manufactured by direct conversion of fibers into fabrics, eliminating the process of yarn manufacturing [48]. Owing to their excellent absorbing and highly porous properties, nonwoven fabrics are among the best options as supporting materials for textile-based hydrogel wound dressings [49].

Cotton fiber is commonly used in various kinds of wound dressings due to its biocompatibility and biodegradability. Cotton is a naturally available, soft, flexible, and durable fiber that exhibits excellent water-absorbing properties due to the presence of hydrogen bonding in the cellulose structure [50,51]. Moreover, cotton fibers are comfortable and friendly to tissues [52]. Therefore, the combination of cotton fiber nonwoven and MCC-based hydrogel is one of the potential choices for wound dressings with medium-to-heavy amounts of exudates. The literature revealed that the TiO_2_-induced cellulose hydrogel–nonwoven cotton fiber has not been developed. In this work, TiO_2_-loaded cellulose hydrogel reinforced with nonwoven cotton was prepared by the sol–gel technique. The prepared composite has a dual advantage due to the combination of cotton fabric with cellulose hydrogel. The fabric acts as support and covers the wound, while cellulose hydrogel can absorb wound exudate and provide a moist environment for rapid healing. Additionally, the loading of TiO_2_ makes the composite antibacterial for wounds. 

## 2. Materials and Methods

### 2.1. Materials

Microcrystalline cellulose (MCC) (purity = 99.5%), titanium oxide (TiO_2_) (purity = 98.6%), sodium hydroxide pellet (NaOH) (purity = 99.9%), calcium chloride dehydrate (CaCl_2_ × 2H_2_O) (purity = 98.9%) and sodium chloride (NaCl) (purity = 99.9%) were purchased from “Daejung Chemicals and Metals Co. Ltd., Siheung-si, Korea”. Cotton fibers were purchased from local market. The cotton and cellulose are biodegradable in nature, whereas titanium oxide is considered as chemically stable and a biocompatible biomaterial.

### 2.2. Nonwoven Cotton Fabric Development

The cotton fibers were opened through the “Toyoda Ohara Blow Room” line. The blow room line consists of sequences of machines containing a bale opener, fine opener, and condenser. The opened fibers were collected for nonwoven web development. Then, the opened fibers were subjected to the fiber opening machines, which fed the fibers toward the carding and cross-lapping machine. A lab-scale, needle-punching machine was used for the nonwoven fabric formation. The fibers in the web were entangled with the needle-punching technique operating at 100 strokes/min and delivered the fabric at a delivery speed of 0.9 m/min. The GSM (g/m^2^) (weight of one square meter fabric in gram) of the developed nonwoven fabric was 150.

### 2.3. Development of Cellulose Hydrogel

**Figure 1:**
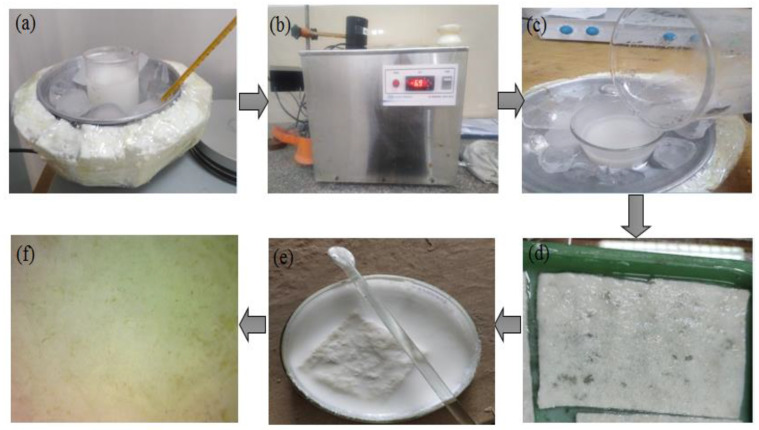
The process flow of cellulose hydrogel reinforced with nonwoven cotton: (**a**) MCC solution preparation at 5 °C; (**b**) NaOH solution preparation at −6 °C; (**c**) mixing of NaOH/MCC solution; (**d**) nonwoven fabric dipped in MCC hydrogel; (**e**) sample dip in TiO_2_ solution; (**f**) cellulose hydrogel-reinforced with nonwoven cotton fabric.

An aqueous solution of 6 wt.% microcrystalline cellulose (MCC) containing 6 g MCC and 94 g water was prepared by stirring it at 5 °C for 2 h. Another aqueous solution of 7.6 wt.% NaOH was prepared by stirring at −6 °C for 2 h. NaOH is an effective and low pollution solvent to dissolve cellulose by using its low concentration in water at low temperatures. Both solutions (MCC and NaOH) were mixed at −6 °C and continuously stirred in a mixed solution for 2 h. After 2 h of stirring at −6 °C, the cellulose hydrogel was developed. The process flow of cellulose hydrogel reinforced with nonwoven cotton is shown in Figure 1.

A homogeneous aqueous solution of TiO_2_ (10 wt.%) was prepared by stirring it at room temperature for 1 h. The developed nonwoven cotton fabric was cut in 5 × 5 cm dimensions. The fabric was dipped in the cellulose hydrogel at room temperature for 1 h. After that, hydrogel, reinforced with nonwoven cotton, was dried in an oven at 50 °C for two hours. The hydrogel was crosslinked with the nonwoven when heated at 50 °C. Then, the washing of composite with the copious amount of distilled water was carried out, which helped to check the regeneration and removal of sodium base. The pH value of the prepared composite in water was obtained in a range of 7.1, which confirmed the release of NaOH in water. A sample of hydrogel reinforced with nonwoven cotton fabric before drying was dipped in TiO_2_ solution for 24 h at room temperature. Thereafter, samples were dried in an oven at 50 °C for 2 h.

### 2.4. Characterization

Three types of samples—the simple nonwoven fabric (CN), hydrogel reinforced with cotton nonwoven fabric (CNHG), and TiO_2_-loaded cellulose hydrogel reinforced with nonwoven cotton (TiO_2_CNHG)—were characterized for a range of properties, as described below.

#### 2.4.1. Scanning Electron Microscopy Analysis

Morphological analysis of hydrogel reinforced with nonwoven cotton was characterized by using a scanning electron microscope (SEM) (Quanta FEG 250, FEI Asia). The samples were coated (3 to 4 nm) with gold before testing. The morphology of pure nonwoven cotton and cellulose hydrogel-incorporated nonwoven cotton were analyzed visually from the SEM images.

#### 2.4.2. Fourier Transform Infrared Spectroscopy (FTIR) Analysis

The presence of hydroxyl group, TiO_2_ groups in the samples were characterized by using FTIR spectroscopy from wave number 4000 to 400 cm^−1^ at transmittance mode.

#### 2.4.3. Mechanical/Comfort Properties Analysis

A tensile strength tester was used to determine the tensile strength and elongation of the prepared samples. This tensile testing was performed according to ASTM D5035-19. To analyze the comfort behavior of prepared composite for wound dressing, the air permeability test was performed on an air permeability tester “SDL-ATLAS, China” according to ASTM D737. A testing area of 20 cm^2^ and the operating pressure of 100 Pascal were selected for the air permeability test. Moreover, the moisture management was analyzed with an SDL-ATLAS moisture management tester (MMT) according to the standard test method of AATCC 195. In this method, MMT drops a 0.9% saline solution drop on the 3 × 3 cm fabric surface and measures its moisture management characteristics.

#### 2.4.4. Exudate-Absorbing Characteristics

The wound exudate absorption analysis was carried out according to EN 13,726-1:2002. A 2.298 g sodium chloride (NaCl) and 0.368 g calcium chloride dehydrate (CaCl_2_ × _2_H_2_O) were dissolved in one-liter water and heated to 37 °C.

**Figure 2:**
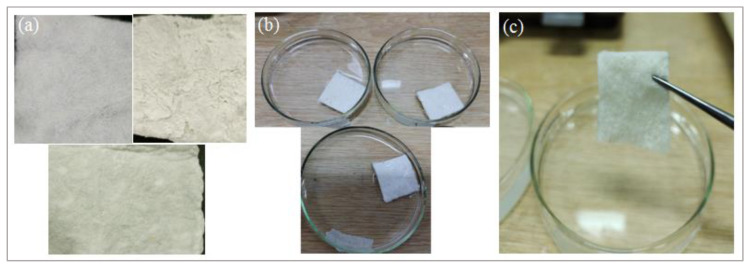
Presentation of exudate-absorbing characteristics: (**a**) developed samples; (**b**) samples inside the fluid; (**c**) excessive fluid removal.

The developed three composite samples, with the dimensions of 5 × 5 cm, were weighed and put into their Petri dishes, as shown in Figure 2. The solution was spread uniformly on the composite samples, and their Petri dishes were placed in an oven at 37 °C. After two hours, the samples were hung for 30 sec for the removal of the extra amount of fluid. Then, wet samples were weighted, and fluid absorptive was calculated according to the following expression:

Fluid absorptive (%) = (wet weight of composite − dry weight of composite)/(dry weight of composite) × 100

The same procedure was repeated for 24 h.

#### 2.4.5. Antibacterial Assay

An agar disk diffusion method was employed for the assessment of the antibacterial activity of the developed composite. An American type *Staphylococcus aureus* was used, which is a Gram-positive and most abundant microbe of the skin flora. It is well known for its association with skin and soft tissue infections [53]. *Staphylococcus aureus* of culture number 6538 bacteria was grown overnight in a nutrient broth solution at 35–37 °C temperature and continuously stirred at 150 r.p.m. All developed samples were sterilized at 121 °C in steam for 15 min. Then, the samples were placed in an agar disk and put inside an incubator for 18 hours. Afterward, the antibacterial properties of all samples were visually analyzed from agar disk images. The antibacterial activity experiment was performed in darkness.

#### 2.4.6. TiO_2_ Particle Size Analysis

**Figure 3:**
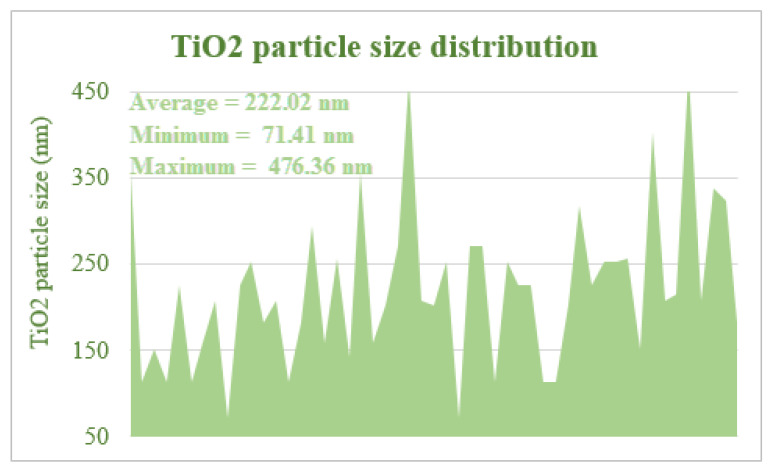
TiO_2_ particle size distribution.

The particle size of TiO_2_ particles on the developed composite was by Image.J software. The average particle size on the composite was 222.02 nm as presented in Figure 3. Moreover, TiO_2_ mass in the composite was 0.118 g (10%), which was determined by gravimetric analysis.

## 3. Results and Discussion

### 3.1. Morphology by Scanning Electron Microscopy (SEM)

**Figure 4:**
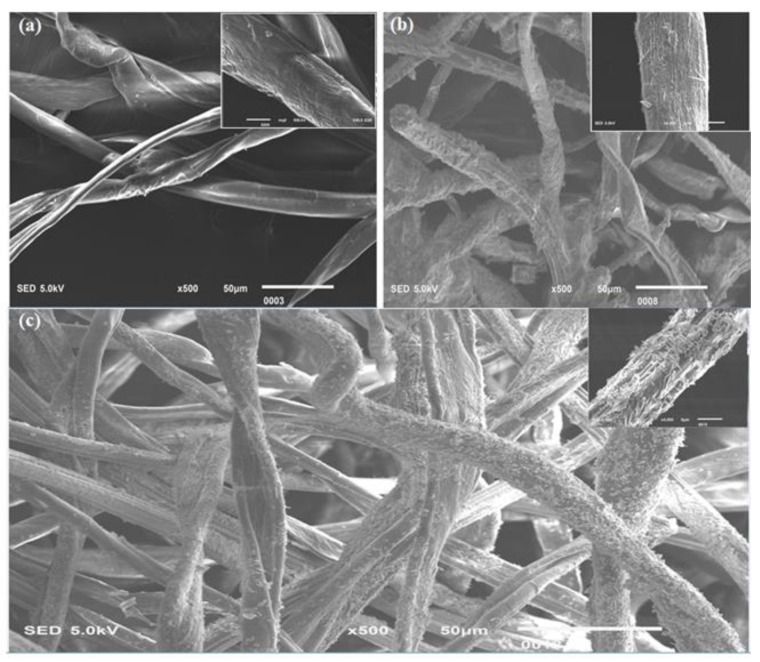
Morphological characteristics of the developed wound dressings: (**a**) pure nonwoven cotton fabric; (**b**) cellulose hydrogel reinforced with nonwoven cotton fabric; (**c**) TiO_2_-loaded cellulose hydrogel reinforced with nonwoven cotton...Figure 4a shows the entanglement of pure cotton, which was achieved by the needle-punching technique. Figure 4b provides evidence for the presence of cellulose hydrogel on the surface of cotton fibers. Figure 4c shows the presence of TiO_2_ particles on the nonwoven cotton cellulose hydrogel composite. This prepared composite has large spaces between the fibers to absorb a high amount of water and wound excaudate. The hydrogel layer is mostly formed on the surface of fibers which has a great capacity to absorb fluids.

Morphological characteristics of the developed samples were analyzed by SEM. The SEM images of pure nonwoven cotton fabric, cellulose hydrogel reinforced with nonwoven cotton fabric, and TiO_2_-loaded cellulose hydrogel reinforced with nonwoven cotton are shown in Figure 4a–c, respectively. 

**Figure 5:**
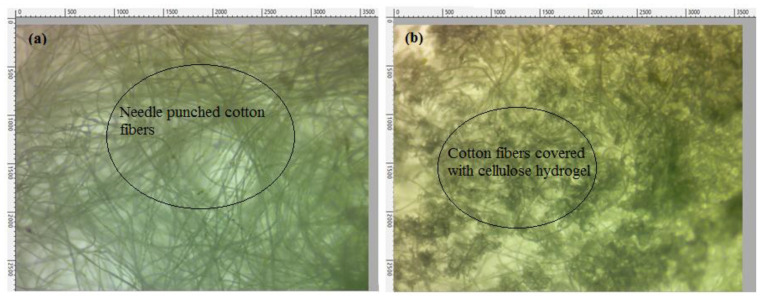
Microscopic images showing the surface appearance of (**a**) pure cotton nonwoven and (**b**) cellulose hydrogel reinforced with cotton nonwoven fabric.

Microscopic images were also taken to analyze the surface appearance of cellulose hydrogel– nonwoven cotton composite. Figure 5a shows a microscopic image of the pure nonwoven cotton fabric, and Figure 5b presents the cellulose hydrogel reinforced with nonwoven cotton fabric.

The images show that pure nonwoven cotton and cellulose hydrogel–nonwoven cotton composite have smooth dirt-free surfaces, and no torn or broken hydrogel or cotton fibers pieces were seen.

### 3.2. Fourier Transform Infrared Spectroscopy (FTIR) Analysis

**Figure 6:**
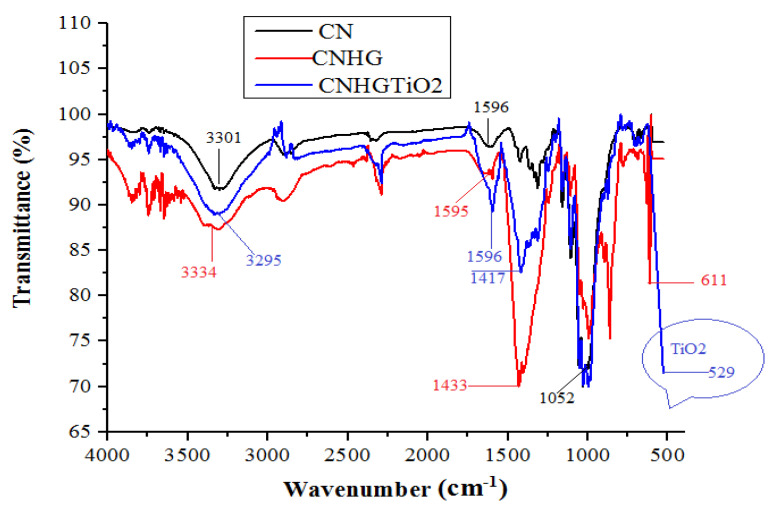
FTIR spectrum of CN,) CNHG, and CNHGTiO_2._

The FTIR spectra of CN, CNHG, TiO_2_CNHG samples are shown in Figure 6.

The absorption peaks occurring at 3301 cm^−1^ and 3295 cm^−1^ confirmed the presence of the –O–H group of cotton and MCC [54]. The absorption peaks of –O–H stretching were observed from 3295 cm^−1^ to 3334 cm^−1^ when the CNHG sample was coated with TiO_2_. The absorption peaks occurred at 1052 cm^−1^ and 1051 cm^−1^, confirming the presence of a strong –C–O group of cellulose [55]. The peaks occurring at 529 cm^−1^ in the CNHGTiO_2_ sample are because of the presence of the TiO_2_ functional group [56].

### 3.3. Air Permeability (AP)

**Figure 7:**
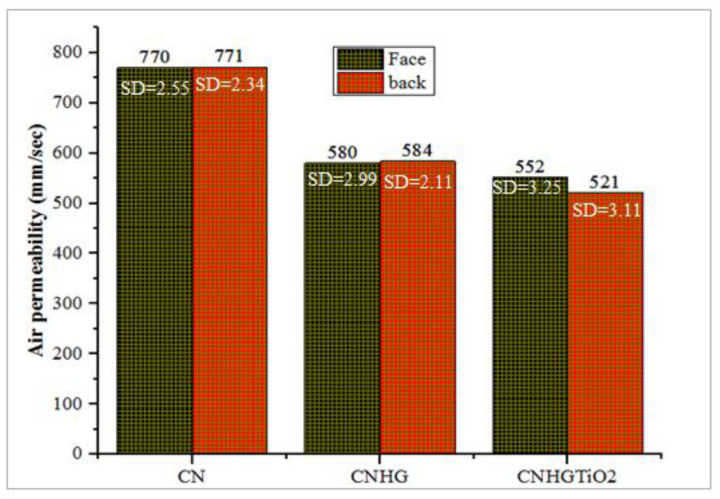
The comparison of various materials for air permeability (face/back) values (mm/sec).

Nonwoven fabrics possess excellent porous structures containing a high volume of air. Nonwoven fabrics have excellent air permeability properties, owing to their porous structure. Air permeability of needle-punched nonwoven fabrics depends on GSM, punch density, and porosity of the structure [57]. The air permeability (Face/Back) values of the CN, CNHG, and CNHGTiO_2_ samples are shown in Figure 7.

The pure cotton nonwoven fabric has an air permeability value of 770 mm/sec, as compared with the MCC-reinforced fabric, with an air permeability value of 580 mm/sec. These results determined that the macropores of nonwoven fabrics were filled with cellulose hydrogel, which reduced air permeability [58]. The reduction in air permeability was caused by the formation of hydrogel into the structure of nonwoven cotton. The results also showed that in CNHG coated with TiO_2_ solution, air permeability decreased to a value of 552 mm/sec. The analysis revealed that TiO_2_ penetrated the open pores of the CNHG samples.

### 3.4. Moisture Management (MMT)

Moisture management property (MMT) is the measurement of dynamic liquid transportation, in multidimensional textile substrates. Moisture management properties of the developed samples are given in Table 1.

The top wetting time of CN, CNHG, and CNHGTiO_2_ was 9.15 s, 2.766 s, 2.203 s, respectively. The results showed that when the MCC hydrogel was applied on the cotton nonwoven fabric, the water absorption time was decreased. This clearly pointed to the high absorption properties of the hydrogel. The top wetted radii of CN, CNHG, and CNHGTiO_2_ were 10 mm, 15 mm, and 15 mm, respectively. This means that the liquid can quickly spread onto the whole surface of the wound dressing and provide a moist environment for rapid wound healing [59].

**Figure 8:**
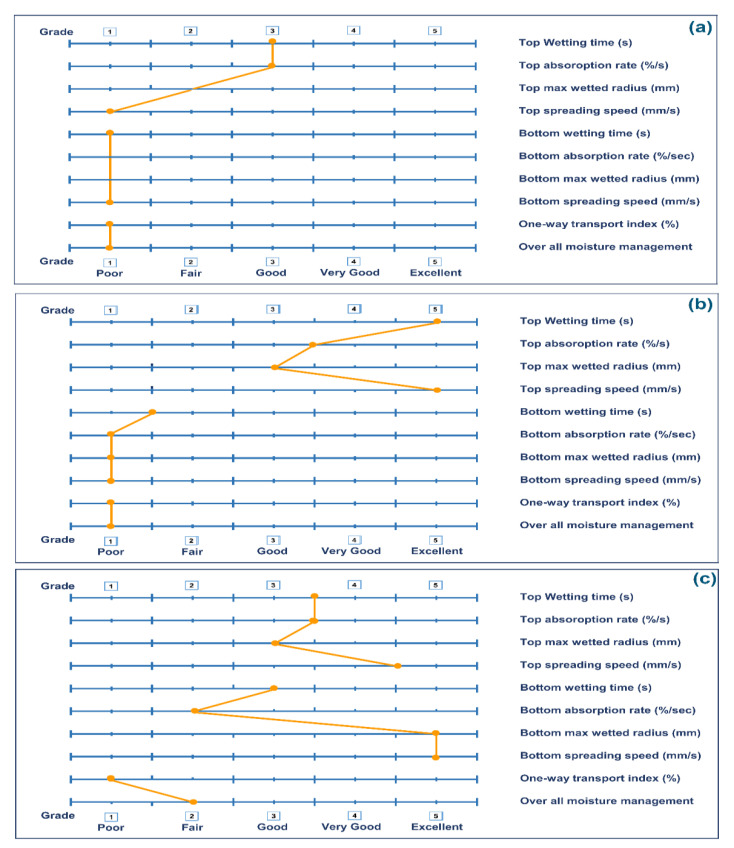
Fingerprints of Moisture management properties of (**a**) CN, (**b**) CNHG, and (**c**) CNHGTiO_2_.

Fingerprints of moisture management properties of CN, CNHG, and CNHGTiO_2_ are shown in Figure 8. Figure 8a shows that the top wetting time and absorption rate of the CN sample fell in a good grade, while Figure 8b,c for CNHG and CNHGTiO_2_ samples indicates that they fell in excellent to a very good grade. The top spreading speed (mm) of the CN sample fell in poor grade, while CNHG and CNHGTiO_2_ fell in excellent grade. The bottom absorption rate of CN, CNHG, and CNHGTiO_2_ fell poor to fair grade. This result indicated that when the developed wound dressing absorbed exudate from the wound area, it retained it, not transferring it to the outer surface. This provides a leakage-proof wound dressing for medium-to-high exudate-releasing wounds.

### 3.5. Tensile Strength

**Figure 9:**
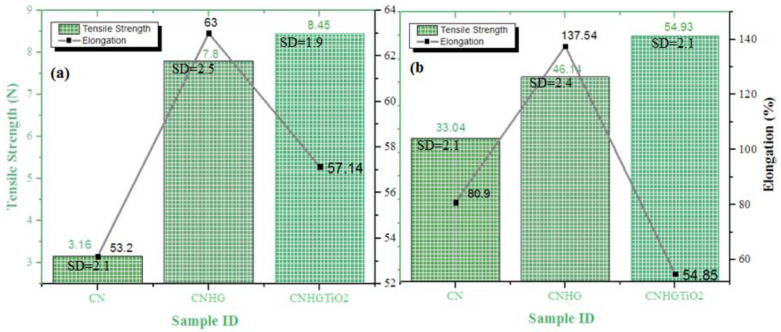
Tensile strength/elongation properties of (**a**) CN, CNHG, and CNHG TiO_2_ dry samples, and (**b**) CN, CNHG, and CNHG TiO_2_ wet samples.

The mechanical strength and elongation of CN, CNHG, and CNHGTiO_2_ samples are shown in Figure 9. CN, CNHG, and CNHGTiO_2_ have a dry tensile strength of 3.16 N, 7.8 N, and 8.45 N, respectively, and a dry form elongation of 53.2%, 63%, and 57.14%, respectively. Moreover, CN, CNHG, and CNHGTiO_2_ have a wet tensile strength of 3.3.04 N, 46.14 N, and 54.93 N, respectively, and wet form elongation of 80.9%, 137.54%, and 54.85%, respectively.

The tensile strength of the CNHG was higher than CN samples owing to the strong hydrogen bonding between cotton fiber and cellulose hydrogel. Moreover, when CNHG was coated with TiO_2_, the tensile strength was further increased, from 7.8 N to 8.45 N. TiO_2_ particles act as a coating on the composite surface. This coating may alter the mechanical properties of the surface; for example, it slightly increased surface rigidity. The TiO_2_ particles were entrapped between cotton fibers, which hindered the moment of fibers. These particles helped to share the external load, which resulted in higher strength [60]. Moreover, when CN was coated with cellulose hydrogel, elongation increased, but when CNHG was coated with TiO_2_, elongation decreased due to minor rigidity of the TiO_2_ layer.

### 3.6. Wound Exudate-Absorbing Characteristic Analysis

**Figure 10:**
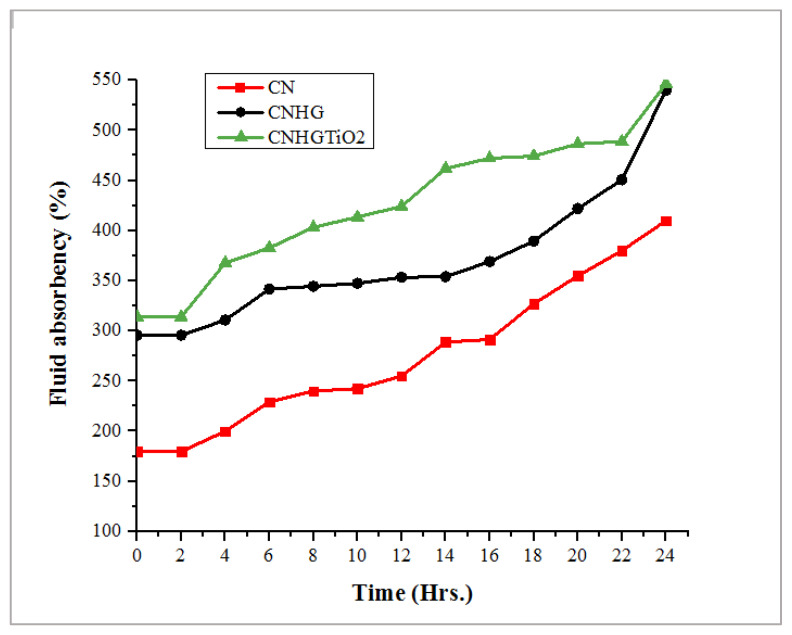
Wound exudate-absorbing properties vs. time of CN, CNHG, and CNHGTiO_2_ samples.

Wound exudate absorption is the primary factor considered while designing wound dressings for the provision of an adequately moist environment. The fluid absorbency % age calculations are given in Table 2, and wound exudate-absorbing characteristics vs. time for CN, CNHG, and CNHGTiO_2_ samples are shown in Figure 10. CN, CNHG, and CNHGTiO_2_ samples have the maximum fluid absorbency % age of 410%, 540%, and 547%, respectively.

The results indicated that when cellulose hydrogel was applied to the nonwoven cotton fabric, the fluid absorbency was increased by 31.7%. This increase was due to the strong hydrophobic characteristics of the cellulose hydrogel.

The results showed that the developed hydrogel had a strong capability for absorbing and retaining water, and it also had an adequate capacity for wound exudate absorption. This moist environment can help the rapid wound healing process [61].

### 3.7. Antibacterial Properties

**Figure 11:**
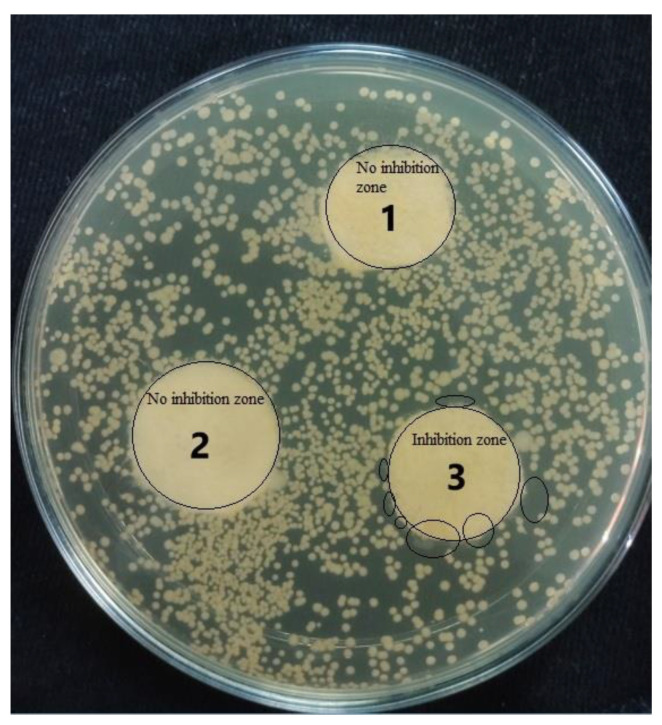
Antibacterial properties of developed three samples.

Antibacterial properties were determined by the agar disk diffusion technique. The antibacterial characteristics of CN, CNHG, and CNHGTiO_2_ are shown in Figure 11. TiO_2_ mass in the composite was 0.118 g (10%), which was determined by the gravimetric method.

It was observed that CN and CNHG samples did not inhibit the growth of bacteria, but the CNHGTiO_2_ sample inhibited bacterial growth. Although the TiO_2_-loaded composite was not highly antibacterial, it demonstrated a moderate-to-low level of antibacterial activity, which was due to growth inhibition and the bactericidal effects of direct contact [62,63,64,65]. It was observed that no colony of bacteria was formed around and on the surface of the TiO_2_-loaded composite.

## 4. Conclusions

The sol–gel technique can be used to develop composites of cellulose hydrogels reinforced with nonwoven cotton fabrics for wound dressing applications. The TiO_2_ coating on the surface of fabricated composite materials yielded good antibacterial activity. The air permeability of the hydrogel composite was decreased as the hydrogel occupied the pores present in the nonwoven cotton. Due to the strong hydrophilic characteristics of hydrogel, the mechanical strength, absorptive capability, and moisture management of the fabricated composite increased, compared with simple nonwoven cotton fabric. In addition, the strong hydrated structure of the cellulose hydrogel–nonwoven cotton composite may provide a moist environment for an enhanced wound healing process. Moreover, cellulose-based composite materials are completely biodegradable, sustainable, and environmentally friendly for several biomedical applications. Therefore, the developed cellulose hydrogel nonwoven composite has an absorptive fibrous hydrogel layer and promising potential for applications for medium-to-high exudate wounds.

## Figures and Tables

**Table 1:** Moisture management properties of the developed three samples.

Sample ID	Wetting Time Top (sec)	Wetting Time Bottom (sec)	Top Absorption Rate (%/sec)	Bottom Absorption Rate (%/sec)	Top Max Wetted Radius (mm)	Bottom Max Wetted Radius (mm)	Top Spreading Speed (mm/sec)	Bottom Spreading Speed (mm/sec)
CN	9.156	119.95	49.39	0.0	10	0.0	0.684	0.0
SD	3.2	2.8	2.99	3.8	2.56	2.1	2.21	2.5
CNHG	2.766	23.166	81.21	3.75	15	0.0	4.653	0.0
SD	3.5	3.33	2.98	2.89	2.51	3.4	3.33	3.4
CNHGTiO_2_	2.203	4.43	55.016	18.51	15	30	6.40	5.44
SD	2.11	2.22	3.1	3.3	2.5	2.1	2.6	3.3

**Table 2:** The fluid absorbency % age calculation of the developed three samples.

Time (hrs.)	CN Wt. (mg)	CNHG Wt. (mg)	CNHGTiO_2_ Wt. (mg)	Fluid Absorbency % Age (CN)	Fluid Absorbency % Age (CNHG)	Fluid Absorbency % Age (CNHGTiO_2_)
0	100	211	215	180	296	314
2	279.7	835	890	180	296	314
4	300	867	1006	200	311	368
6	329.2	932	1038	229	342	383
8	340	938	1082	240	345	403
10	342.5	944	1104	243	347	413
12	355.1	957	1127	255	354	424
14	388.9	958	1208	289	354	462
16	391.5	990	1230	292	369	472
18	427	1033	1235	327	390	474
20	455	1101	1261	355	422	487
22	480	1162	1266	380	451	489
24	510	1350	1390	410	540	547
Average	361.45	944.46	1080.92	275.38	370.54	426.92
SD	104.70	258.63	291.65	74.88	67.97	70.14
CV%	28.97	27.38	26.98	27.19	18.34	16.43

## Data Availability

Not applicable.
